# High-Energy Ejection of Molecules and Gas-Dust Outbursts in Coal Mines

**DOI:** 10.3390/e23121638

**Published:** 2021-12-06

**Authors:** Sergii D. Kaim

**Affiliations:** Faculty of Electrical Engineering, Automatic Control and Computer Science, Opole University of Technology, Ul. Prószkowska 76, 45-758 Opole, Poland; s.kaim@po.edu.pl; Tel.: +48-77-537-809-228

**Keywords:** high-energy phenomena, boundaries of liquid stability, ejection of atoms and molecules, self-acceleration of ejection, gas-dust outbursts, dissociation on shock wave fronts, self-ignition of hydrogen

## Abstract

In the current work, using the framework of the formalism found in the Bogolyubov–Born–Green–Kirkwood–Yvon (BBGKY) equations for the distribution functions of particle groups, the effective single-particle potential near the surface of the liquid was analyzed. The thermodynamic conditions under which a sudden opening of the liquid surface leads to high-energy ejection of atoms and molecules were found. The energies of the emitted particles were observed to be able to significantly exceed their thermal energy. Criteria of the ejection stability of the liquid surface and the self-acceleration of ejection were formulated. The developed theory was used to explain the phenomenon of the self-acceleration of gas-dust outbursts in coal mines during the explosive opening of methane traps. The results also explained the mechanisms of generating significant amounts of methane and the formation of coal nanoparticles in gas-dust outbursts. The developed approach was also used to explain the phenomenon of the self-ignition of hydrogen when it enters the atmosphere.

## 1. Introduction

In condensed systems, there exist high-energy phenomena whose characteristic energies are measured by several electron volts or more per particle. Such phenomena include shock and detonation waves, gas-dust outbursts in mines, and sonoluminescence, among others. A characteristic feature of high-energy phenomena in condensed systems is that they occur in thin layers near an open surface or at the interface. At the same time, atoms and molecules in the surface layers play a decisive role in all activation processes. The characteristic energies of such particles far exceed the thermal energies of the particles and their binding energies in the bulk of the materials. Therefore, the state of such activated particles can be described as corresponding to a liquid or gas state. The phenomenon of the ejection of particles near the phase boundary in high-energy phenomena is a decisive factor determining the kinetics of such phenomena.

For the development of microelectronics on a nanometer scale, the mechanisms of the phenomena of ejection from condensed systems at the level of individual atoms, molecules are important. The kinetics of the ejection processes of individual particles determine the limits of technological processes. Therefore, studying the forces acting on individual atomic particles is topical. Ejection processes also determine the boundaries of macroscopic stability on the surface of condensed systems.

The atomization phenomenon is most often understood as the dispersion of a liquid onto microdrops. To clarify the mechanisms of high-energy ejection phenomena, it is important to study the behavior of individual atoms and molecules of liquids in the near-surface layer and their ejection into the environment. The description of high-energy phenomena using the equations of a continuous medium has significant limitations arising from the assumptions made in obtaining such equations. At the level of individual atoms and molecules, equations of a continuous medium can be considered asymptotic of more fundamental equations of statistical physics.

The phenomenon of high-energy ejection can be described in terms of a single-particle potential that acts on individual atoms and molecules in a condensed medium and beyond. Such a potential is the result of all pair and multiparticle interactions of particles and their correlations with the selected particle. This potential can correspond to both attractive and repulsive forces depending on the thermodynamic state of the condensed medium.

To describe high-energy ejection, the behavior of a single-particle potential near the surface of the medium is important. The best conditions for the manifestation of forces acting on an individual particle can be created by abruptly opening the surface of the medium and allowing particles to be emitted from the surface layer. There is significant experimental material on the study of the phenomenon of spontaneous combustion of hydrogen during its sharp release into the air. High-energy phenomena accompany the abrupt opening of methane traps in coal using explosive mining methods in coal mines.

The ejection of atoms and molecules from a liquid can be described in terms of the work function [1]. The characteristic distances at which the ejection of fluid particles occurs correspond to nanometer values. The behavior of atoms or molecules under conditions of high-energy phenomena near the phase boundaries can be described in terms of the effective single-particle potential created by the collective particles of the system [1,2].

Among high-energy phenomena, there are distinguished phenomena whose characteristic feature is ejection from the open surface of a liquid. Of considerable interest are spontaneous self-ignition upon discharge from a pressure vessel into the atmosphere (hydrogen) and the self-amplification of gas-dust emissions during the sharp opening of methane traps in coal mines. The mechanisms of high-energy phenomena are determined by physicochemical processes with individual molecules and atoms in the nanometer surface layer. High kinetic energies of the emitted atoms and molecules correspond to ejection phenomena.

An urgent technological problem involves the safe storage of hydrogen and monofuels. The phenomenon of the self-ignition of hydrogen upon release into the atmosphere is an obstacle to its widespread use in the energy sector. The conditions of the self-ignition of hydrogen are widely studied both experimentally and theoretically. A description of various cases of the self-ignition of hydrogen, an analysis of the experimental conditions of various authors, and an analysis of the proposed mechanisms of the phenomenon are presented in a review [3]. The reverse Joule–Thomson effect, electrostatic charge generation, diffusion ignition, sudden adiabatic compression, and ignition from a hot surface were also analyzed. However, even after review, the mechanism of the phenomenon was not completely clear [3].

The conditions of the self-ignition of hydrogen, natural gas, mixtures of hydrogen and methane compressed in a pipe with a sharp opening of the diaphragm, and their release into the air were studied at different pressures, geometries, and sizes of installation [4,5,6,7,8,9,10].

One commonality among all the reviewed studies was the strong dependences of the self-ignition pressure of hydrogen on the diameter of the pipe and the existence of a delay time for self-ignition. No self-ignition was observed for methane [5]. A numerical study regarding hydrogen self-ignition in the framework of a model, including the gas-dynamic transport of viscous gas and the kinetics of hydrogen oxidation and heat transfer, was carried out in [4,6], and indicated hydrogen ignition as a result of the formation of a shock wave. A shock wave formed in front of the gaseous hydrogen propagating in the pipe.

The most general macroscopic approach to the dynamics of a multicomponent liquid or gas requires the joint solution of a system of continuity equations for each component, an equation for the momentum of the mixture, an equation for the internal energy, and expressions for the viscous stress tensor and the heat flux vector, which are the sum of the contributions due to thermal conductivity and diffusion. The numerical implementation of the solution of such a system of equations for multicomponent fluids and complex geometric structures was given in [11,12]. A numerical study of the self-ignition of compressed hydrogen upon release into the air using program [12] was carried out in [7]. It was assumed that ignition occurred if the temperature exceeded 1500 K and the mole fraction of OH was more than 0.001. For the ignition criteria, it was also assumed that these two parameters could not fall below the specified limit values until the end of the simulation. The possibility of the formation of a shock wave obtained in numerical gas-dynamic modeling upon the release of compressed hydrogen into the air indicated high-energy phenomena. However, the microscopic nature of the high-energy phenomena of atomization and self-amplification at the hydrogen–air interface remains unexplored.

Another example of the phenomenon of high-energy atomization, which leads to catastrophic macroscopic phenomena, is related to gas and coal outbursts in coal mines. The phenomena of gas and coal outbursts in coal mines, as a result of their consequences, remain the most dangerous gas-dynamic phenomena encountered in underground work. Gas and coal outbursts are characterized by large amounts of gas, crushed coal, and nanometer-sized coal dust. Outbursts vary widely, ranging from a few tons to tens of thousands of tons. A classic example is the outburst at the Yu.A. Gagarin mine in the Donetsk region (15 July 1969, depth of 710 m) with up to 14,500 tons of coal and methane volume—according to various estimates—from 250,000 to 900,000 m^3^. The outburst time was 32 s. The area of the hole through which the outburst of gas-dust flowed was equal to 5 m^2^. The outburst covered tens of meters per second [13,14,15,16,17]. The gas pressure in the coal mass during the opening was 5 MPa [18]. As a result of this discharge, a crosscut 650 m long was completely covered with coal. Moreover, the thickness of fine coal dust was the maximum of all known cases and reached 40–50 cm [16].

The outbursts’ parameters indicated a high-energy mechanism operating at the molecular level (the formation of free methane, the generation of ultrafine coal particles, the short-term effects). Gas and coal emissions are similar to the Bridgman phenomenon in condensed systems [19]. Under the conditions of high pressures and shear deformation in condensed matter, dispersion and high-velocity ejection can occur (Bridgman effect) [19]. This causes the generation of ions, high-energy electrons, and photons, in addition to the explosive destruction of solids on Bridgman anvils that lasts for $10^{-5}-10^{-7}s$ with high-speed (200–2000 m/s) outbursts [20]. The Bridgman effect has been observed for almost all substances.

An important characteristic of coal is its methane content. This content varies over a wide range and can reach values of 50 m^3^/t. Statistical processing of the conditions of more than 30,000 emissions led the authors of the work [21] to conclude that the minimum methane content, which acts as one of the irreplaceable conditions for possible outbursts of coal gas, was 8 m^3^/t. The analysis of the largest gas and coal outbursts indicated the generation of significant volumes of methane, which reached values up to 500 m^3^/t of the coal outburst, and sometimes even more than 1000 m^3^/t [22,23]. The source and, accordingly, the mechanism for generating significant volumes of free gas in a short emission time (seconds) remains a mystery. During release, a certain mechanism of gas generation was at work, forming gas in a free state. At the same time, the volume of free gas was ten times higher than the volume of methane, which was in a bound state in coal (adsorbed, absorbed, dissolved) [21].

Coal mines in Ukraine have recorded more than 11 thousand outbursts [18,24]. For the period 1951–2005, 7230 outbursts of coal and gas were recorded, of which 22% were sudden, with shaking explosions in 68% and with the remote control of machines and mechanisms in 10% of cases [18,24].

A sudden release of coal and gas brings finely dispersed coal from the face to the mined-out space. A distinctive feature of coal and gas emissions is the presence of finely ground—even down to the nanoscale—coal, the mass of which can be from 20% to 50% of the total outburst mass [25]. Despite the abundance of papers discussing possible mechanisms for outbursts of coal and gas, the mechanism for generating coal dust remains unknown.

One of several dangerous and common phenomena leading to the formation of an explosive gaseous environment in mine workings is the sudden pulsed emission of methane during roof collapse in the zone of influence of geological disturbances [26,27]. Pulse emissions of methane occur spontaneously. In case of gas breakthroughs from the roof, methane emission can increase 6–35 fold and reach maximum values of up to 77 m^3^/min for a short period of time. Then, over a period of hundreds of hours, a gradual decrease in the flow rate of the evolved gas occurs.

There are several hypotheses regarding the possible nature of pulsed methane emission [26,28]. However, the main mechanism of pulsed methane release is thought to be the release of gas from gas traps (pockets) [26,28,29]. The existence of gas traps requires an explanation of the mechanism of gas isolation at the boundaries of traps. The area surrounding the abundant gas zone must have low permeability to prevent high-pressure gas loss through the coal seam itself [28].

There are many theoretical approaches to explain the mechanism of gas and coal outbursts. All of the following explanations have been classified in a review [30]. There are two main approaches that describe the mechanisms of outbursts of gas and coal. The first is an approach that postulates the existence of pockets (traps) containing volumes of soft or crushed coal enclosed in cracked coal. According to the hypothesis, these form reservoirs of free gas contained in the voids of the cracks. These volumes of crushed coal are associated with fault or shear zones. If a mine develops closer to the soft coal area, explosive gas and coal outbursts may occur [30,31]. The second approach (focused on dynamic theories) postulates that the volume of highly gaseous, cracked coal, which is subjected to heavy loads during mining, is subject to outburst. As production approaches such a volume of coal, coal cracks are destroyed with the release of desorbed gas, which leads to an outburst [30]. The assumption of the existence of cracked coal with a high gas content, which is able to quickly desorb gas when the compression pressure is relieved, is common to both explanations [30].

The development of theoretical concepts of gas and coal outbursts in the framework of macroscopic continuum theories (mechanics, hydro- and gas dynamics) over the 20 years following the work published in [30] did not lead to an explanation of their mechanism and causes [18,23,28,31,32]. Two mysterious phenomena remain unexplained: (1) methane generation in quantities significantly superior to the methane in coal and (2) the dispersion of a significant part of the emitted coal at a nanoscale [25]. Despite the wide variety of conditions under which gas and coal ejections occur, there is a universal part of these fast and high-energy phenomena associated with the generation of large quantities of gas and fine coal.

The work of [33] provides a classification system according to the energy scale of gas dynamic phenomena in coal mines based on the analysis of gas and coal outbursts. The authors considered a range of outbursts, from very weak outbursts (with energies below 10^4^ J) to outbursts with energies above 10^9^ J—which correspond to earthquake energies [33].

Physicochemical conditions leading to gas and dust outbursts are being investigated from many different directions. An important part of that research focuses on identifying the properties and structure of coal, as well as the characteristics of such states that would indicate the possibility of gas and coal emissions [31]. However, reliable predictors of gas and coal outbursts have not been found. It has been established that the causes of gas and dust outbursts are related to the porous coal structure. The cracked-porous structure of coal ranges from nanometers to millimeters. The saturation of the cracked-porous structure with methane strongly depends on the degree of coal metamorphism and varies within the range of 5 to 50 m^3^/t of coal [13]. Coal methane may be present in the form of a solid solution formed by methane molecules in pores with sizes on the order of molecules [14].

Studies performed by IR spectroscopy showed that, in the fragmented coal formed during the sudden outburst, destruction occurred at the molecular level, including a large number of methyl groups detaching during the outburst [34]. Studies of changes in the carbon ratio in aromatic and aliphatic molecular components of coal before and after gas-dust outbursts indicated significant changes in the molecular structure of coal. After outbursts, the number of methyl groups in the aliphatic component of coal was significantly reduced. At the same time, there was no change in the benzene component [34].

The NMR spectra investigated the ${}^{13}C$-NMR spectra of coal before and after gas-dust outbursts [14]. Significant changes in the NMR -spectrum, corresponding to the separation of light hydrocarbon molecules, were recorded. The number of aliphatic bonds in coal changed as a result of gas-dust outbursts, i.e., gas-dust outbursts were the processes of molecular bond-breaking [14]. Breaks in molecular bonds during the passage through the front of coal destruction were associated with the separation of methyl groups, and macroscopic outburst parameters indicated a high-energy detonative nature of the phenomenon of gas and coal outbursts.

Another unsolved problem is a lack of clarity regarding the mechanisms of generating dust and gas outbursts. Since the outbursts are explosive, a possible solution to this problem is related to the mechanism of initiating detonation processes in condensed energetic materials. The dissociation energy of organic molecules—with the separation of the methyl group $CH_{3}$ and the breaking of the C−C bond—changes depending on the structure of the molecules in a fairly wide range of 3÷4.4 eV [35]. The energy required for the separation of the hydrogen atom from the methane molecule is 4.44 eV, and the average binding energy of C−H in the methane molecule is 4.25 eV. The separation energy of the $CH_{3}$ group from coal molecules is on the order of 3÷4 eV, and the separation energy of the hydrogen atom is on the order of 4 eV [36]. The energy of processes at the microscopic level, which can lead to the dissociation of coal molecules to form methyl groups and hydrogen (which form methane molecules), remains unclear.

The scale of dissociation energies of coal molecules of 3÷4 eV formally corresponds to thermal processes with temperatures on the order of tens of thousands of degrees. However, such high temperatures with gas and dust outbursts have not been recorded. This means that the physicochemical transformations at the front of gas and dust outbursts must have a non-equilibrium impact character. This character of the propagation of the gas-dust outburst front is possible when a compression wave is formed with a sharp front, similar to a shock wave, at the front of which is the shock dissociation of coal molecules. Significant changes in the molecular structure of coal, without a significant increase in temperature, are only possible in the shock wave process. The main feature of this process is the destruction of coal and the transition from the loaded state to a mixture of gas and highly dispersed coal. In general, this process corresponds to the shock wave of unloading.

Weak shock waves of unloading in the framework of macroscopic theory were first investigated in the work of [37,38]. The condition of their existence was determined to be the inequality ${\left(\partial^{2}v/\partial p^{2}\right)}_{S}<0$, where v,p are the volume and pressure of the gas, respectively. The derivative is calculated at steady entropy. This condition can be fulfilled near the critical point of the liquid in the gas phase in a small section of values of pressure and volume. Experimental confirmation of the existence of weak rarefaction shock waves was obtained in [39]. The relatively low critical parameters of methane ($T_{c}=190.55\;K,$ $p_{c}=46.41\cdot 10^{5}\;\mathrm{Pa},$ $v_{c}=0.006161\;m^{3}/\mathrm{kg}$), compared to temperatures and reservoir pressures in coal mines, led to the conclusion of the supercritical state of methane in coal seams. Therefore, the theory of weak shock rarefaction waves in liquids near a critical point cannot plausibly be applied to self-sustaining gas and dust outbursts. Currently, there is no macroscopic theory of strong rarefaction shock and detonation waves.

The generation of significant volumes of methane and highly dispersed coal to a nanoscale indicates the high-energy phenomena of coal atomization during gas and dust outbursts in coal mines. Therefore, the study of the mechanism of gas-dust ejections requires a microscopic approach at the atomic-molecular level to describe the phenomenon of the atomization of hydrogen atoms and methyl groups from the structure of coal.

In this work, a microscopic approach to the phenomena of gas-dust outbursts in coal mines and the spontaneous combustion of hydrogen during its sharp release into the atmosphere, based on the works [1,2], was developed. The possibility of the generation of a compression wave in a gas due to the phenomenon of the self-acceleration of the front of an unloading wave during a sharp opening of a volume with gas was analyzed. Such conditions arose inside a methane trap when it was suddenly opened as a result of the use of explosives. Self-acceleration of the unloading front was accompanied by an increase in pressure at the system boundary due to reactive forces acting on the system in the surface layer during the ejection of molecular particles with superthermal velocities.

The origin of the unloading shock wave at the beginning of gas-dust outbursts was possibly related to condensed methane in a sharply open trap. The main role in this mechanism was played by the ejection of methane molecules. At the leading front of the shock wave, methane molecules were emitted in the direction of the front movement, causing the shock dissociation of the coal molecules and generating methyl groups and hydrogen atoms. At the trailing front of the shock wave, methane molecules were emitted and supported the structure of the compression wave when they were ejected. The number of generated methane molecules was a critical quantity for self-sustaining the destruction of the molecular structure of coal and the formation of methane and coal nanoparticles.

## 2. Effective Single-Particle Potential in Semi-Limited Liquid

We studied the possible sources of high-energy monatomic processes in a nonhomogeneous liquid. We postulated the existence of a simple nonhomogeneous fluid whose density gradient was directed perpendicular to the plane of the liquid surface. The interaction of atoms was described by the pair potential of the interatomic interaction. The Hamiltonian of such a system can be written in the form:

(1) $$H=\sum_{i=1}^{N}\frac{P_{i}^{2}}{2M}+\frac{1}{2}\sum_{i\neq j=1}^{N}\Phi \left(R_{i}-R_{j}\right),$$

where N is the number of atoms in a non-uniform liquid; $P_{i},M$ is the momentum and mass of atoms; and $\Phi \left(R\right)$ is the interaction energy of two atoms.

The unary distribution function in a nonhomogeneous fluid satisfies the first equation of the Bogolyubov–Born–Green–Kirkwood–Yvon chain of equations [1,2,40].

(2) $$k_{B}T\frac{\partial }{\partial z}F_{1}\left(z\right)+\frac{1}{v_{0}}\int d^{3}R_{1}F_{2}\left(z,z_{1},\rho^{∥}\right)\frac{\partial }{\partial z}\Phi \left(z,z_{1},\rho^{∥}\right)=0,$$

where $k_{B},T$ is the Boltzmann constant and the temperature; $v_{0}$ is the volume per one atom in a liquid; $F_{2}\left(z,z_{1},\rho^{∥}\right)$ represents the pair function of atom distribution; and $\rho^{∥}=R^{∥}-R_{1}^{∥}$ is the component of the difference of vectors parallel to the surface whose equation z=0.

The pair distribution function $F_{2}\left(z,z_{1},\rho^{∥}\right)$ can be represented as:

(3) $$F_{2}\left(z,z_{1},\rho^{∥}\right)=\;F_{1}\left(z\right)F_{1}\left(z_{1}\right)g\left(z,z_{1},\rho^{∥}\right),$$

where $g\left(z,z_{1},\rho^{∥}\right)$ is the pair correlation function of the fluid. Then, Equation (2), with respect to representation (3), can be written in the equivalent integral form:

(4) $$F_{1}\left(z\right)=\exp\left[-U\left(z\right)/k_{B}T\right],$$

where $U\left(z\right)$ plays the role of a monatomic self-consistent potential in the Boltzmann distribution and effectively acts on individual atoms [1,2].

(5) $$U\left(z\right)\;=\;-\frac{1}{v_{0}}\int_{-\infty }^{z}dz_{1}\int d^{3}R_{2}F_{1}\left(z_{2}\right)g\left(z_{1},z_{2},\rho_{12}^{∥}\right)\frac{\partial \Phi \left(R_{12}\right)}{\partial R_{12}}\frac{z_{2}-z_{1}}{R_{12}}.$$

Substituting Equation (4) into the right-hand side of (5), we obtain, for the monatomic potential, a nonlinear Hammerstein integral equation [41].

(6) $$U\left(z\right)\;=\;-\frac{1}{v_{0}}\int_{-\infty }^{z}dz_{1}\int d^{3}R_{2}\exp\left[U\left(z_{2}\right)/k_{B}T\right]g\left(z_{1},z_{2},\rho_{12}^{∥}\right)\frac{\partial \Phi \left(R_{12}\right)}{\partial R_{12}}\frac{z_{2}-z_{1}}{R_{12}}.$$

Similarly, if we substitute the Equation (5) in the right-hand side of Equation (4), then we obtain the Hammerstein equation for the unary distribution function $F_{1}\left(z\right)$.

(7) $$F_{1}\left(z\right)\;=\;\exp\left[\frac{1}{k_{B}Tv_{0}}\int_{-\infty }^{z}dz_{1}\int d^{3}R_{2}F_{1}\left(z_{2}\right)g\left(z_{1},z_{2},\rho_{12}^{∥}\right)\frac{\partial \Phi \left(R_{12}\right)}{\partial R_{12}}\frac{z_{2}-z_{1}}{R_{12}}U\left(z\right)\right].$$

The exact solutions of Equations (6) and (7) are, at the time of this writing, not available. Their representation would correspond to a generalized solution to the problem of the structure of a liquid surface. Since we are interested in a monatomic potential in the surface layer of a semi-infinity fluid, we use Equation (5), choosing model expressions for the unary distribution function, the pair correlation function, and the interaction energy of a pair of atoms. Formally, this approach corresponds to the first iteration when solving the integral Equation (6).

Later in the model calculations, we used the Fowler approximation [42,43]. Accordingly, for a density profile in a semi-bounded fluid, we took a stepped profile, and for the correlation function $g\left(z,z_{1},\rho_{12}^{∥}\right)$, we used the approximation as follows:

(8) $$g\left(z,z_{1},\rho_{12}^{∥}\right)≅F_{2}\left(\left|R_{1}-R_{2}\right|,n\right),$$

where $F_{2}\left(\left|R_{1}-R_{2}\right|,n\right)$ is the pairwise function of the distribution of atoms in a homogeneous liquid with the density of the number of atoms n.

In the approximation (8), the expression for the effective monatomic potential (5) is written as:

(9) $$U\left(z\right)=-\frac{1}{v_{0}}\int_{-\infty }^{z}dz_{1}\int d^{3}R_{2}F_{1}\left(z_{2}\right)F_{2}\left(\left|R_{1}-R_{2}\right|,1/v_{0}\right)\frac{\partial \Phi \left(R_{12}\right)}{\partial R_{12}}\frac{z_{2}-z_{1}}{R_{12}}.$$

On the basis of potential (9), we calculated the effective monatomic potential in a non-uniform liquid with a postulated density distribution. The fluid density distribution was chosen stepwise with a unary distribution function $F_{1}\left(z\right)\;=\;\Theta \left(-z\right)$, where $\Theta \left(z\right)$ is a stepwise Heaviside function.

For model calculations of the properties of shock-compressed methane, the model potential of Lennard–Jones with the parameters ε=144 K and σ=3.796 Å, taken from [44], were used

(10) $$\Phi \left(R\right)=4\varepsilon \left[{\left(\sigma /R\right)}^{12}-{\left(\sigma /R\right)}^{6}\right],$$

in addition to using the Weeks–Chandler–Andersen (WCA) approximation [45,46,47] for a pairwise molecular distribution function.

**Figure 1:**
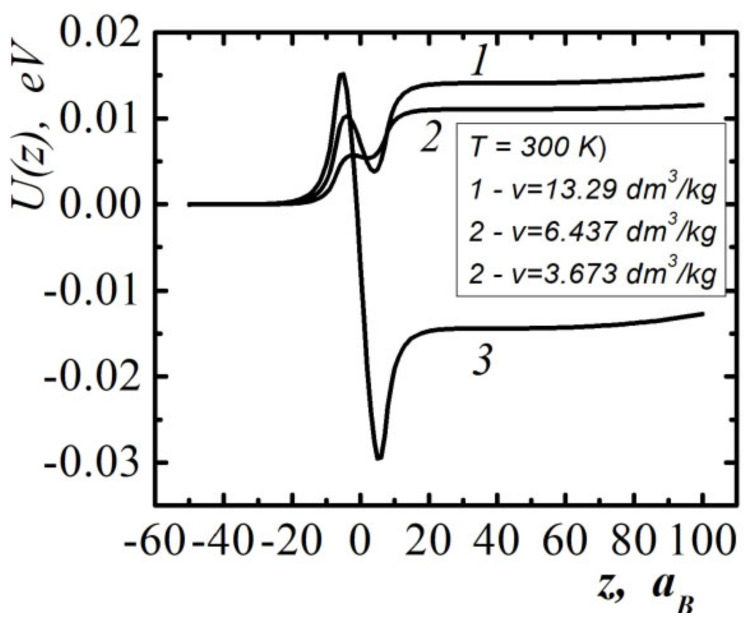
One-particle potential near the surface of the semi-bounded methane (z<0) at temperature T=300K, different values of the specific volume. $a_{B}$ is the Bohr radius.

Figure 1 show the dependencies of the one-particle potential acting on methane molecules in the surface layer (methane occupies half-space z<0) at three different values of specific volume and temperature T=300K. As the volume decreases, the nature of the one-particle potential changes from attracting the individual molecules (curves 1 and 2) to the ejection (curve 3).

**Figure 2:**
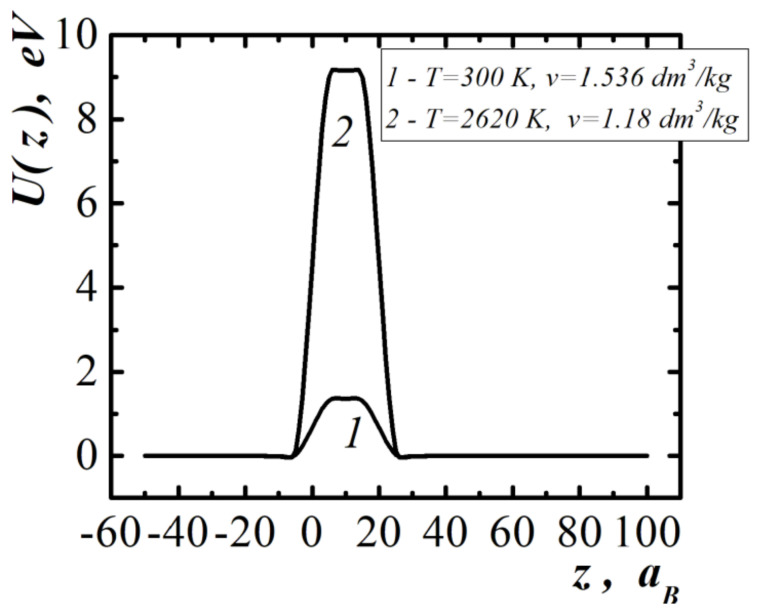
One-particle potential near the surface of a layer of compressed methane with thickness $d=20a_{B}$.

The dependences of the one-particle potential acting on the molecules in the layer of compressed methane, with a thickness $d=20a_{B}$ for two values of temperatures and specific volumes, are shown in Figure 2. The one-particle potential is ejective in relation to individual molecules. Curve 2 in Figure 2 corresponds to the work function of molecules from the interlayer, sufficient for shock dissociation of molecules at pair collisions (degree of dissociation). The scales of values of one-particle potentials shown in Figure 1 and Figure 2 are significantly different.

**Figure 3:**
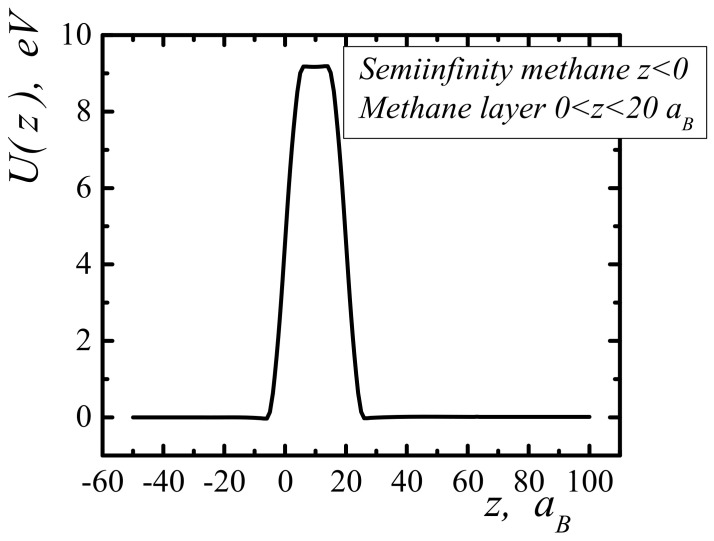
One-particle potential near the surface of semi-bounded methane and layer of compressed methane with thickness $d=20a_{B}$. Temperature and specific volume of semi-bounded methane: $T=300\;K,\;v=6.437\;dm^{3}/\mathrm{kg}$, compressed methane layer—$T=2620\;K,\;v=1.18\;dm^{3}/\mathrm{kg}.$

Therefore, in the case of semi-bounded methane and the layer of compressed methane in contact with it (Figure 3), the resultant one-particle potential differs little from the potential of the layer of compressed methane (Figure 2). To be able to realize the shock dissociation of molecules at the shock wave front, a prerequisite is the possibility of the self-enhancement and self-acceleration of the initial compression wave. This effect is possible with the sharp (impact) opening of the methane trap.

The compressed layer of methane at the front of the shock wave played a double role. On the one hand, the emission of molecules in the direction of wave propagation led to the shock dissociation of molecules at the front. On the other hand, the emission of molecules in the opposite direction provided the self-support of the shock wave. The output of a shock wave of sufficient intensity from methane to coal can ensure the dissociation of coal molecules and the separation of methyl groups and hydrogen from the molecules of coal.

## 3. Conditions of Self-Acceleration of Unloading Waves under the Opening of a Methane Trap

The general equation of the state of a homogeneous liquid, the molecules of which interact with the help of paired central forces, can be written in terms of the release of molecules from a liquid in a vacuum [1,2]:

(11) $$p=n\left(k_{B}T-A_{l-v}/2\right),$$

where n is the density of the number of molecules; $k_{B},T$ are Boltzmann and the absolute temperature; and $A_{l-v}$ is the work function of molecules from a semi-infinity fluid with a flat surface into a vacuum,

(12) $$A_{l-v}=\frac{4\pi }{3v_{0}}\int_{0}^{\infty }dR\frac{\partial \Phi }{\partial R}g_{0}\left(R\right)R^{3},$$

$\Phi \left(R\right)$ is the interaction energy of two molecules; $g_{0}\left(R\right)$ is the pair distribution function of molecules in a homogeneous liquid; and $v_{0}$ is the volume per molecule. With given values of pressure p, density of number of molecules n, and temperature of liquid T by means of (11), it is possible to calculate the work function $A_{l-v}$.

(13) $$A_{l-v}=2\left(k_{B}T-p/n\right)$$

It follows from Equation (13) that, at certain values of thermodynamic parameters, the work function of the molecules can become negative. In the case of a sharply open surface of a liquid with such thermodynamic parameters, molecules can be emitted by the surface. The velocity of the emitted molecules can be estimated from the equality of the kinetic energy of the mass M molecule and the module of work function.

(14) $$Mu^{2}/2=\left|A_{l-v}\right|.$$

It follows from relations (13) and (14) that the velocity of the emitted molecules can differ significantly from their thermal velocities.

Consider the reaction forces of the flux of emitted molecules to a liquid-upon-rapid unloading of a compressed semi-bounded fluid [41]. The pressure $p^{\prime }$, corresponding to the reactive force at one-dimensional flow of fluid at a density ρ with velocity u, can be written as:

(15) $$p^{\prime }=\rho u^{2}$$

or subject to (14):

(16) $$p^{\prime }=Mnu^{2}=2n\left|A_{l-v}\right|,$$

where n is the density of the number of molecules.

The condition of the equality of the pressure in the liquid and the pressure of the reactive force in the emission of molecules from the open surface of the liquid, taking into account the equation of state (11), can be written in the form [41]:

(17) $$A_{l-v}=-2k_{B}T/3$$

The condition of self-acceleration of the emission of molecules from the unloaded surface of a liquid $p^{\prime }>p$ can be written in the form of inequality.

(18) $$A_{l-v}<-2k_{B}T/3$$

Condition (17) corresponds to the boundary line of the self-acceleration (self-support) of the emission of molecules. When performing inequality (18), a self-sustaining compression wave is formed as a result of the emission of molecules from the surface in the surface layer of the liquid. In an extreme case, the compression wave will turn into a shock wave, the front of which will dissociate the molecules of coal hydrocarbons with the release of methyl groups and hydrogen.

**Figure 4:**
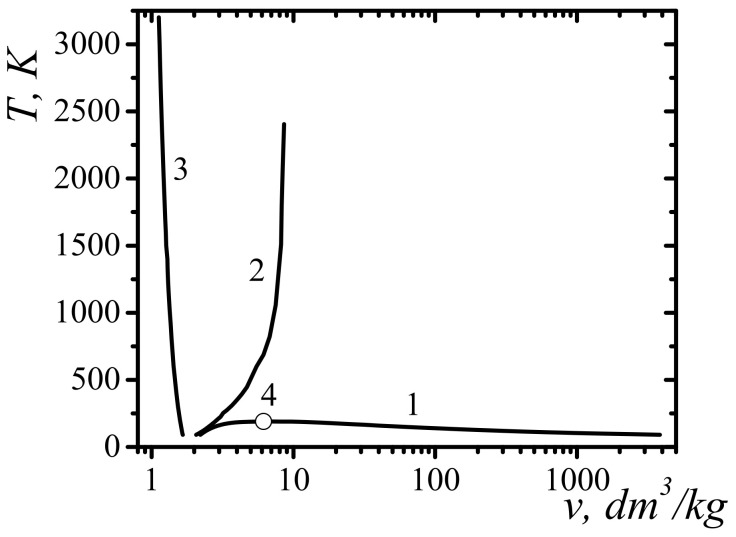
Phase diagram of methane in variables specific volume—temperature (v−T): 1—liquid-gas equilibrium line [47]; 2—boundary line of self-acceleration of emission of molecules; 3—line of maximum compression of the fluid; 4 is the liquid-gas critical point.

In Figure 4, in variables specific volume–temperature v,T shows the position of the self-acceleration line for the emission of methane molecules. In Figure 4, at the parameters of v,T, corresponding to the points to the left of curve 2, the necessary condition for the self-acceleration of emission from the unloaded surface of compressed methane is fulfilled. The effect of the self-acceleration of emission from a rapidly unloaded surface is due to the non-ideality of the system. For an ideal gas for which $A_{l-v}=0$, such an effect is absent.

In Figure 4, curve 3 corresponds to the compression of liquid methane, in which the first minimum of the pairwise distribution function of molecules reaches zero. In the case of greater compression, the pairwise distribution function begins to acquire negative values and it becomes meaningless to apply the method of particle group distribution functions. Therefore, curve 3 in Figure 4 is naturally called the line of maximum fluid compression. Curve 3 in Figure 4 corresponds to the intervals of change in temperature and specific volume $T\in \left[91;\;3200\right]\;K,\;$$v\in \left[1.126;\;1.66\right]$ dm^3^/kg.

**Figure 5:**
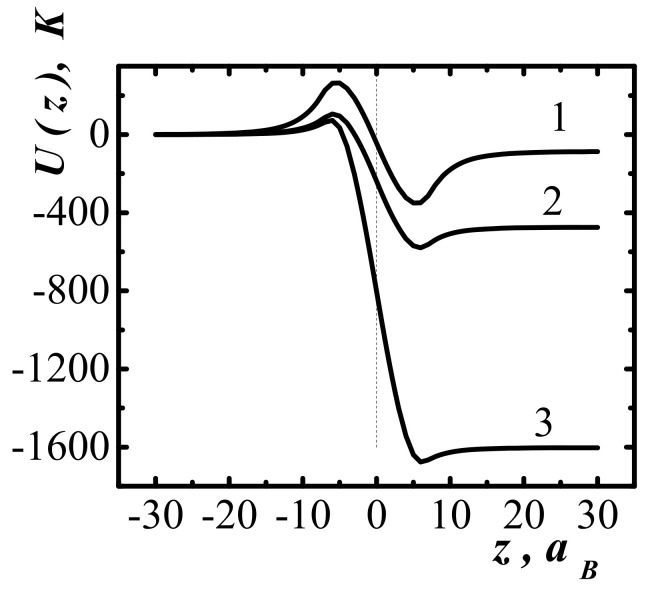
Results of calculations of one-particle potential at the boundary line of the self-acceleration of the emission of methane molecules in Fowler’s approximation with unary distribution function $F_{1}\left(z\right)\;=\;\Theta \left(-z\right)$: curve 1—T = 108.28 K, v=2.208  dm^3^/kg; curve 2—T=687.68 K, v=6.161 dm^3^/kg; curve 3—T=2404 K, v=8.6 dm^3^/kg.

Figure 5 present the dependences of the one-particle potential $U\left(z\right)$ of the mean forces near the methane surface, whose density profile was modeled in accordance with Fowler’s approximation by a step function. The specific volume and temperature were chosen at several points on the emission self-acceleration limit curve (curve 2 in Figure 4). The width of the layer at which the potential $U\left(z\right)$ near the surface changed, is the order of 5 Å. With increasing temperature and specific volume, the width of the layer is decreased. As can be clearly seen from Figure 5, the one-particle potential of the average forces with respect to individual molecules was ejective. This means that the surface was unstable with respect to the emission of individual molecules. The near-surface layer, in which a sharp decrease in potential was localized, played the role of an accelerator to the emitted molecules.

**Figure 6:**
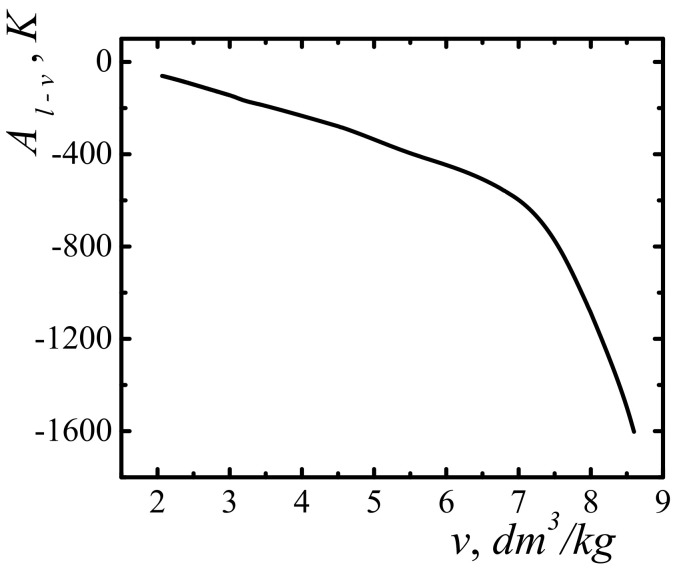
Work function $A_{\;l-v}$ along the self-acceleration boundary line. The temperature changes in the interval $\left[91;\;2404\right]\;K$.

Figure 6 show the dependence of the work function $A_{l-v}$ along the self-acceleration boundary line as a function of the specific volume. The graph in Figure 6 corresponds to the temperature variation interval [91; 2404] K. The calculations indicate the limited top-down values of the volume under which the boundary conditions of the self-acceleration of the emission of molecules can be realized. In the case of methane, for the specific volume of v>8.8 dm^3^/kg, such conditions are not fulfilled under any temperature values.

**Figure 7:**
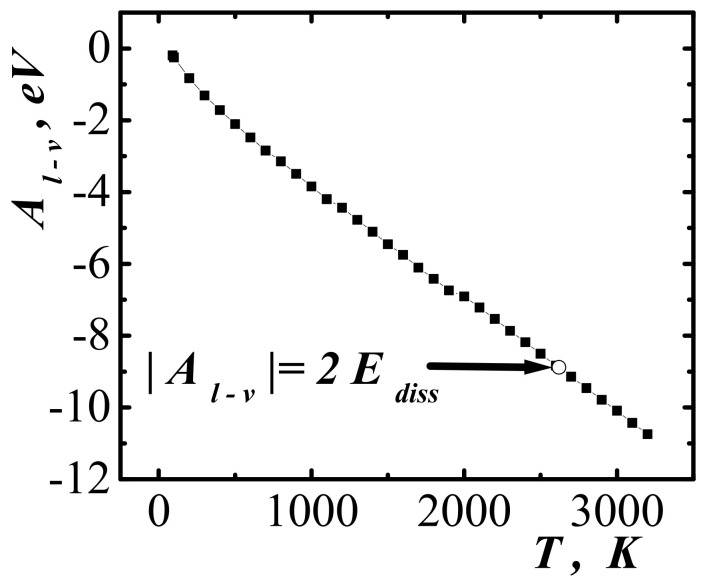
Work function along the line of maximum compression. The temperature changes in the interval $\left[91;\;3200\right]\;K$. The arrow indicates the point at which the condition of homogeneous dissociation $CH_{4}\to CH_{3}+H$ is satisfied.

To determine the degree of compression required to create accelerated molecules that could initiate dissociation, the work function of molecules from semi-bounded methane along the line of maximum fluid compression (curve 3 in Figure 4) was calculated. Figure 7 show the results of the calculation of the work function $A_{l-v}$ along the line of maximum compression of the fluid. The graph in Figure 7 corresponds to the temperature variation interval [91; 3200] K.

For the dissociation of a molecule when pushing a pair of molecules, the minimum required kinetic energy of the relative motion of molecules is equal to twice the dissociation energy [48,49]. Figure 7 show the point corresponding to equality $\left|A_{l-v}\right|=2E_{diss}$, where the dissociation energy of the methane molecule according to the scheme $CH_{4}\to CH_{3}+H$ is found. The thermodynamic parameters corresponding to this point are: temperature T=2620 K, specific volume v=1.165 dm^3^/kg, pressure p=241,024 bar, and the velocity of emitted molecules V=10,336 m/s.

The obtained estimation of the parameters of semi-bounded liquid methane corresponded to the conditions of homogeneous initiation of the process of molecular dissociation. The obtained parameters of the initial shock dissociation of molecules were quite high. Note that the obtained temperature values (in the energy scale), sufficient to initiate the dissociation of molecules at the shock wave front, were much smaller than the values of the molecular dissociation energies. Our calculations demonstrated the outstanding importance of the density and potential energy of the system to the effects of the initiation of chemical reactions. The significant density dependence of the conditions sufficient to initiate dissociation is a manifestation of the large forces of repulsion of molecules at short distances and the correlation effects associated with them.

**Figure 8:**
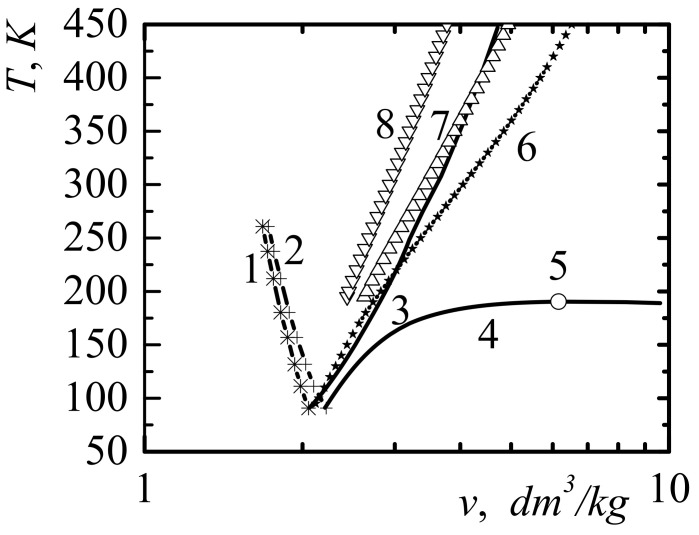
Methane diagram: curves 1 and 2 are equilibrium solid–liquid equilibria (dependences $v_{sol}(T)$ and $v_{liq}(T)$); 3 represents self-support of pressure in a liquid; 4 is the liquid–gas equilibrium line; 5 is the critical point; lines 6, 7, and 8 correspond to isobars at pressures p=400 bar, p=600 bar, p=1000 bar, respectively.

Figure 8 show the volumetric volume-temperature v−T for methane experimental dependences: $T\left(v_{sol}\right)$ for the equilibrium solid–liquid equilibrium—curve 1 [50] ($v_{sol}$—specific volume of solid methane); for the liquid–solid equilibrium—curve 2 ($v_{liq}$—specific volume of liquid methane) [50]. Additionally, this figure shows the calculated boundary line of the self-acceleration of the front of the rarefaction wave, for points of which the equality $A_{l-v}=-2k_{B}T/3$ is satisfied—curve 3; liquid–gas equilibrium line—curve 4 and critical point—5 [47]. Figure 8 also show the lines of isobar for methane, respectively, for pressure p=400 bar—curve 6; for pressure p=600 bar—curve 7; for pressure, p=1000 bar—curve 8. Data for isobars were taken from the book [51].

As shown in Figure 8, the data suggest that the initial condition for the manifestation of the self-acceleration of the rarefaction front with a sharp opening of the methane trap at order temperatures T~300 K is the initial shock compression of methane to the pressure of the order p~500 bar. Such methane compression is possible when opening methane traps with the use of explosions or percussion instruments commonly used in underground work. This condition is facilitated by the fact that the methane in the trap is at pressures equal to, or larger than, 100 bars. It is also clear that a prerequisite for converting a compression wave into a shock wave with an intensity sufficient for the dissociation of hydrocarbon molecules is a sufficient trap size.

## 4. Conditions of Impact Dissociation of Coal Molecules

The possibility of generating methane when the shock wave exits from a methane trap into coal is subject to the fulfillment of the necessary conditions. One of the conditions is the shock dissociation of coal molecules when interacting with the shock wave. To estimate the thermodynamic conditions at the shock wave front, in which the methane molecules with kinetic energy sufficient for shock dissociation of molecules will be emitted in the wave propagation direction, the equation of the state of liquid methane in form (11) was used. Since the dissociation energies of coal molecules in the order of the energy of dissociation of methane molecules, equality was required as an additional condition:

(19) $$A_{l-v}=2E_{diss},$$

where $E_{diss}$ is the dissociation energy of the methane molecule. The dissociation energies of the methane molecule according to the scheme $CH_{4}=CH_{3}+H$ are equal to 4.44 eV [35].

After substitution of condition (19) in (11) we obtain:

(20) $$p=n\left(k_{B}T+E_{diss}\right).$$

Equation (20) describes in variables p, v, T the necessary thermodynamic conditions at the front of the shock wave, under which the shock dissociation of molecules will occur at the front. The choice of dissociation condition (19) corresponds to the shock dissociation of one molecule when two methane molecules collide. Therefore, Equation (20) corresponds to the degree of dissociation α=0.5.

**Figure 9:**
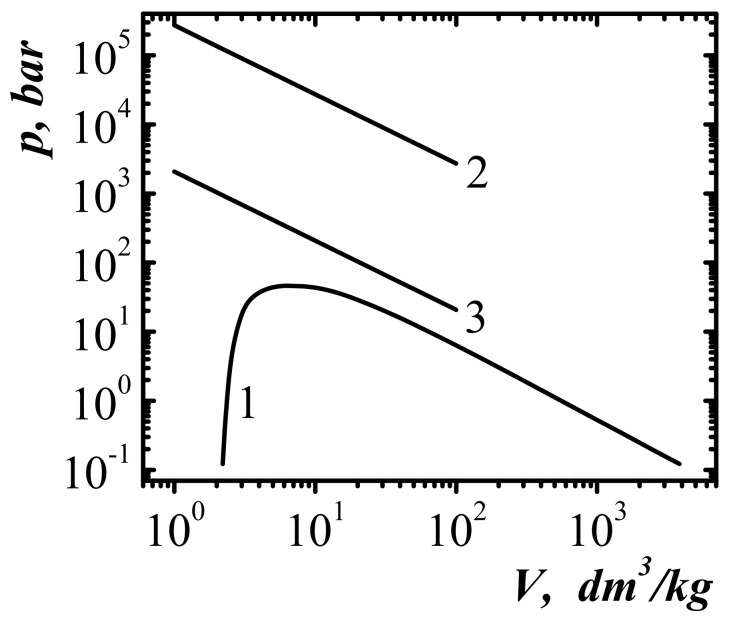
Phase diagram of methane: 1 represents the equilibrium line liquid–gas; 2 represents the line of shock dissociation at the front of a plane shock wave (degree of dissociation α=0.5); 3 is the boundary line of self-acceleration (T=300 K).

In Figure 9, in the phase plane p−v, the liquid–gas equilibrium line for methane is shown, as well as the line of shock dissociation of methane molecules, which was calculated at the choice of $A_{l-v}=2E_{diss}=8.88$ eV and at temperature T=300 K. Equations (19) and (20) correspond to the criterion of the shock dissociation of the molecules in the front of a plane shock wave. Figure 9 also show a boundary line 3 of the self-support and self-acceleration of the rarefaction front of the compressed fluid, which is described by equation:

(21) $$p=4nk_{B}T/3.$$

With thermodynamic parameters corresponding to the points above the line 3, the conditions of the self-acceleration of the rarefaction front are realized.

As can be seen from Figure 9, the conditions of the self-support and self-acceleration of the unloading front (curve 3) are realized at much lower pressure values than the conditions of realization of the phenomenon of shock dissociation (curve 2). Therefore, it is clear that converting the rarefaction front into a rarefaction shock wave of intensity sufficient for shock dissociation of molecules requires a certain length of run.

In nitromethane, the excitation of a detonation by a shock wave transforms it into a detonation wave at a distance of several centimeters [52]. Therefore, one might think that, in liquid methane, the rarefaction wave can reach the intensity of a shock wave sufficient to trigger the dissociation mechanism at distances of the order of several centimeters.

## 5. Instability of the Rarefaction Shock Wave Front and Generation of Coal Nanoparticles

The considered mechanism of the beginning of non-thermal emission can lead to the formation of high-speed molecular streams. Consider the possible macroscopic consequences of the formation of such flows. Among the mechanical manifestations, one should take into account the reaction forces arising from the high-velocity ejection of a part of the system. The reactive force generated by the one-dimensional emission of the substance is a source of additional backpressure in the surface layer of coal (Equation (15)).

**Figure 10:**
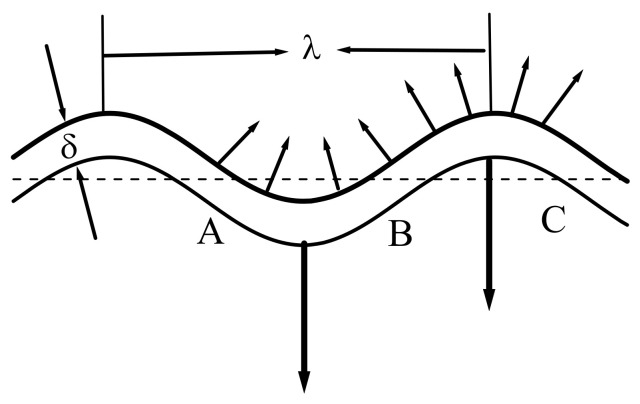
A fragment of the front of the physical reaction of surface release at the initial linear stage of fluctuation.

A spontaneous random fluctuation of pressure from a reactive force in the process of physical surface release will lead to distortion of the plane geometry of the front, which propagates into the coal mass. The convexity of the front, which went forward, is associated with its acceleration, the concavity of the front, which lagged behind (with respect to the initial flat shape), is associated with deceleration (Figure 10).

Since the release of the coal surface occurs mainly along the normal to it (this is the energetically most favorable situation), the fluxes of the ejection particles behind the convex sections of the front will be characterized by convergence, and behind the concave ones will be characterized by divergence (Figure 10). Therefore, in the first case, there will be an increase in pressure in the converging flow behind the front, and in the second, a decrease in the diverging flow. The addition or subtraction of such additional pressure entails an intensification of the process of ejection of the substance of the near-surface layer or its weakening.

**Figure 11:**
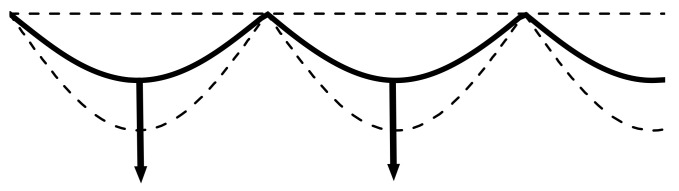
A fragment of the front of the physical reaction of surface release at the nonlinear stage of fluctuations.

In the first case, behind the AB section, this will again lead to a further increase in pressure, and in the second case, to its decrease. The indicated mechanism of positive feedback is similar to that for the propagation of the front of chemical transformation for a normal flame [53,54]. It gives rise to the instability of the front surface release and leads, over time, to pulling forward along the process of its tongues, AB, which accelerates and quickly eats up the parts of the BC that are lagging behind. Thus, when the front propagates into the depth of the coal, the surface release front takes the form of a rosette with the amplitude of its petals growing in time (Figure 11).

Thus, the instability of the physical process of surface release gives rise to a significant distortion of the front and its propagation, increasing the perimeter of the front itself and, consequently, the release of matter, i.e., self-acceleration of the surface release process. The same instability will develop in the other direction of the front, making its own contribution to the self-acceleration of the process and giving the rosette petals a spatial (or bumpy) shape.

The thickness δ of the front of the physical reaction of the release of the coal surface is determined by the length of the zone of significant change in the value of the self-consistent single-particle potential in methane at the microlevel, and is estimated by ~(10÷15) Å. By virtue of the described mechanism of positive feedback, we were able to conclude the following: the shorter the wavelength λ of the disturbances (and the greater curvature of the distorted front), the more intense the convergence and divergence of the flux of the ejected converted matter that will take place in the areas that accelerate and lag behind (Figure 10). Thus, an additional change in pressure in these areas will intensify, contributing to the process of freeing the surface of the coal, intensifying the positive feedback, and increasing the destabilization of the process as a whole.

However, the presence of methane viscosity η in the near-surface layer will impose a limitation from below on the wavelength of unstable perturbations since the viscous forces will exert their stabilizing effect on the development of perturbations. Thus, the greater this viscosity, the more curved the surface release front. Therefore, the instability of the process can be reliably expected only when the inertial forces generated by the flow perturbations will exceed the forces of viscous flow stabilization. The Reynolds number Re=Vλ/ν [53] is a quantitative estimate of the ratio of these forces, where ν is the kinematic viscosity and V is the flow velocity. Thus, the instability of the surface release front can manifest itself only for Re numbers exceeding one. Hence, we obtained a lower estimate for the wavelength $\lambda >\nu /V=\lambda_{\min}$.

To estimate $\lambda_{\min}$, we chose the thermodynamic conditions on the boundary line of the self-acceleration of the emission of molecules, whose Equation (21) can be written in the form:

(22) $$p\;=\;\frac{4k_{B}T}{3v},$$

where v is the specific volume. Note the equation of state for the ideal gas, $p\;=\;\frac{k_{B}T}{v}$. Since the modulus of the work function is equal to the kinetic energy of the molecule, taking into account (13), we obtained $\frac{mV^{2}}{2}=2\left(pv-k_{B}T\right).$ Hence, for the velocity of the emitted molecule, we obtained $V\;=\;2\sqrt{\frac{k_{B}T}{3m}}.$ Note that, for all points on the boundary line of self-acceleration, the velocity of emitted molecules depends only on temperature.

For the estimates, we selected the temperature and specific volume at the boundary line of self-acceleration of the emission of molecules $T\;=\;300\;K,\;v=0.004737\;m^{3}/\mathrm{kg}.$ The rate of emitted methane molecules V=1489 m/s. The required value of the dynamic viscosity was selected from the experimental data for the dynamic viscosity $\eta =111.62\;\cdot 10^{-6}\;N\cdot s/m^{2}$ [51], and the density of methane $\rho =1/v=422.2\;\mathrm{kg}/m^{3}.$ For kinematic viscosity, we obtained $\nu =0.2644\;\cdot 10^{-6}\;m^{2}/s$. Then, $\lambda_{\min}=\nu /V=1.775\cdot 10^{-10}\;m=1.775\;Å\;\approx \;2\;Å$.

All fluctuation perturbation wavelengths λ > 2 Å on the surface of coal in a layer of compressed methane with a thickness δ~(10÷15) Å are unstable. These perturbations can evolve and, at the nonlinear stage, take the form shown in Figure 11. The non-uniform distribution of the pressure field along the two-dimensional surface corresponds to the non-uniform distribution of the work function of the methane molecules directed into the coal (Figure 2 and Figure 3). This will determine, at the nonlinear stage of the development of perturbations, the nanometer size range of dispersed (atomized) coal, which is ejected due to the physical release of the coal surface. Therefore, coal nanoparticles with dimensions λ > 2 Å will be present in the gas-dust outburst.

## 6. Conditions of Self-Ignition of Hydrogen upon Release into Atmosphere

The combustion reaction of hydrogen and oxygen has been comprehensively studied since the 1920s [55,56]. This reaction is an example of a wide class of chain reactions consisting of a series of repeating cycles involving free atoms and radicals. Chain reactions are characterized by three types of transformations: nucleation, branching, and the termination of chains. The reaction has a threshold character. When a chain is nucleated, colliding molecules break up into atoms and radicals, a process that consumes energy. The disintegration of molecules into atoms and radicals occurs when the reaction initiation energy is imparted to the system. After the stage of initiation of the reaction and the appearance of radicals and atoms, the reaction develops spontaneously [55,56].

To study the initial stage of the spontaneous combustion of hydrogen when it is suddenly released into the air, collisions of hydrogen molecules with air molecules—which lead to the production of atoms and radicals H, O, OH with their subsequent participation in chain reactions—are important [57,58,59]. Possible decays and the production of atoms and radicals via collisions of molecules have the following form (the values of threshold energies are indicated in parentheses):

(23) $$H_{2}\;\to H+H\;(4.44\;\mathrm{eV}),$$

(24) $$O_{2}\;\to O+O\;\left(5.12\;\mathrm{eV}\right),$$

(25) $$H_{2}\;+\;O_{2}\;\to \;2(OH)\;(0.69\;\mathrm{eV}).$$

Among the indicated schemes of endothermic reactions (23)–(25), the energetically most favorable is (25). After the shock formation of radicals OH, they participate in subsequent reactions with the formation of atoms and radicals H, O, OH (the continuation and branching of the chain) [59].

The shown schemes (23)–(25) are realized in inelastic collisions corresponding to bimolecular reactions

(26) $$A_{1}+A_{2}\to A_{3}+A_{4}.$$

where $A_{i}$ are molecules in some states.

The total kinetic energy of molecules in inelastic collisions changes:

(27) $$Q=(T_{3}+T_{4})-(T_{1}+T_{2}),$$

where Q is the reaction energy. For exothermic reactions, Q>0, for endothermic reactions Q<0, for elastic collisions Q=0.

The kinetic energy of the reaction products must be positive, i.e., $T_{3}+T_{4}>0$. From the law of conservation of energy, we obtained $T_{1}+T_{2}+Q>0$. If the reaction is endothermic, then this inequality is fulfilled under the condition

(28) $$T_{1}+T_{2}>\left|Q\right|.$$

In the case of reaction (25), the following inequality must be fulfilled: $T_{H_{2}}+T_{O_{2}}>\left|Q\right|,$ where $\left|Q\right|=0.69\;\mathrm{eV}.$ The kinetic energy of an oxygen molecule is chosen equal to the average kinetic energy of molecules at temperature T=300 K, i.e., $T_{O_{2}}=3k_{B}T/2\;=0.08\;\mathrm{eV}.$ We chose the kinetic energy of a hydrogen molecule $T_{H_{2}}$, equal to the modulus of the work function of the molecule from hydrogen ($A_{H_{2}}<0$), i.e., $T_{H_{2}}=\left|A_{H_{2}}\right|.$ Then the condition for initiating process (25) was written $\left|A_{H_{2}}\right|+3k_{B}T/2>\left|Q\right|$, or

(29) $$\left|A_{H_{2}}\right|>\left|Q\right|-3k_{B}T/2=0.61\;\mathrm{eV}.$$

The resulting condition (29) can be written in terms of thermodynamic parameters p, T, n if we take into account the equation of state (13). Equality

(30) $$\left|A_{H_{2}}\right|=\left|Q\right|-3k_{B}T/2$$

**Figure 12:**
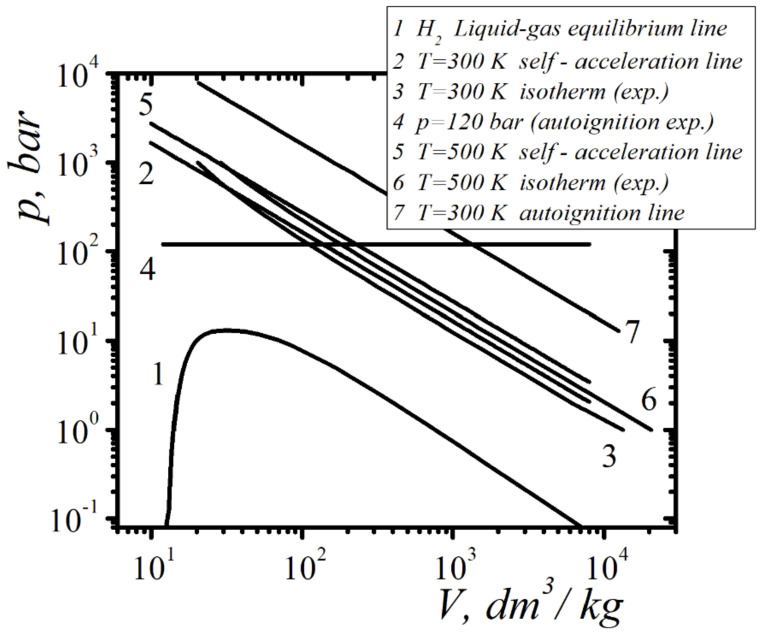
p−V phase diagram of hydrogen: 1: liquid–gas equilibrium line; 2: emission self-acceleration line T=300 K; 3: isotherm T=300 K; 4: line of self-ignition of hydrogen when released into the air (experiment) p=120 bar; 5: emission self-acceleration line T=500 K; 6: isotherm T=500 K; 7: self-ignition boundary line.

In the planes of different pairs of thermodynamic parameters corresponds to the boundary lines of self-ignition of hydrogen when it is suddenly released into the air. In Figure 12, in variables p, V, the boundary line of self-ignition corresponds to curve 7.

Figure 12 in variable pressure—specific volume p−V for hydrogen, show the results of calculating the position of self-acceleration boundary lines for the emission of molecules at different temperatures. It also shows experimental dependences $p\left(V\right)$ of isotherms for the same temperatures. Isotherms and fluid–gas equilibrium lines were constructed on the basis of experimental data taken from [47]. The self-acceleration boundary lines of emission and the isotherms have intersection points, which indicate the existence of thermodynamic conditions under which the sharp opening of the fluid leads to the formation of a compression wave in the liquid due to the action of the reaction force in the emission of molecules. This effect is of great importance for many unresolved high-pressure gas storage tasks. Even with minor leakage of the hydrogen from the vessel at high pressures into the atmosphere, its self-ignition, and subsequent explosions, occur. Of all the cases of inflammation reported in [3], 85% were left unexplained. The self-ignition of hydrogen in the air occurs at temperatures above 536 °C.

Figure 12 also show the limit of the self-ignition of hydrogen that has leaked into the atmosphere at room temperature, corresponding to the pressure of the 120 bar (horizontal line), found in experiments [3]. Achieving the self-ignition of hydrogen at room temperature is not possible without self-accelerating leakage. The effect of the self-acceleration of the emission of liquid molecules in this paper explains the achievement of hydrogen self-ignition conditions. The phenomenon of the self-acceleration of the emission of molecules by a liquid surface can lead to the transformation of a compression wave into a shock wave propagating inside a liquid. At the shock wave front, under conditions of self-acceleration of emission of molecules, the conditions of the shock dissociation of molecules can be achieved.

When a vessel with compressed hydrogen is suddenly opened, which has thermodynamic parameters corresponding to the boundary line of self-acceleration, the high-energy emission of molecules leads to the self-acceleration of the ejection and the initiation of a rarefaction shock wave. As a result, there is an increase in the emission of molecules, which leads to a change in the parameters at the front of the rarefaction shock wave. Further self-acceleration of the ejection will lead to the achievement of self-ignition by hydrogen at the shock front of the state corresponding to the boundary line (curve 7 in Figure 12). The transition of hydrogen at the front of the rarefaction shock wave from the state at the boundary line of self-acceleration to the state at the boundary line of self-ignition takes some time. This time is the delay time of hydrogen autoignition, which was observed in the experiment (the time interval between the moment the vessel is opened and the moment of autoignition). A theoretical estimate of the autoignition delay time requires solving the corresponding kinetic problem.

## 7. Discussion of the Results

In this work, using the BBGKY equations of formalism for particle group distribution functions, the effective one-particle potential near the liquid surface was analyzed. The performed model calculations of the effective one-particle potential in the broad ranges of thermodynamic parameters indicated the possibility of realizing the ejection character of this potential for individual molecules in the surface layer of fluid. This kind of ejection one-particle potential corresponds to the phenomenon of molecular atomization from the surface of a liquid. The one-particle potential is due to pairwise interparticle interactions and correlations and depends on the thermodynamic parameters of the system. The magnitude of changes in the one-particle potential can reach tens of eV, which corresponds to high-energy phenomena in condensed systems.

The stability limits of liquids with respect to high-energy ejection from the surface were also investigated. When the molecules are emitted to the surface of the liquid, reactive forces act, which can create a pressure greater than the pressure inside the liquid. The analytical criterion of the self-acceleration and self-support of the emission of molecules were obtained. The effects of the self-acceleration and self-support of molecule emission leads to the generation of a compression wave and its transformation into a rarefaction shock wave (Figure 2 and Figure 3).

This paper investigated the necessary conditions for the implementation of gas and dust outbursts in coal mines. One of the necessary conditions found was the condition of self-support and self-acceleration of the phenomenon of emission of molecules from the sharply exposed surface of liquid methane. The paper showed the possibility of the realization of shock waves of rarefaction due to the emission of molecules. The positions of the self-support boundary lines of the phenomenon of the emission of methane molecules on the phase planes v − T and p−v were calculated.

This work also dealt with the problem of the generation of coal nanoparticles with high-energy emissions of coal and methane. The nanometer sizes of dust particles are associated with the instabilities of fluctuation perturbations at the front of the rarefaction shock wave entering the coal mass. Taking viscosity into account leads to a lower bound on the lengths of unstable fluctuation perturbations and thereby determines the minimum sizes of generated coal nanoparticles.

In contrast to existing ideas regarding the possibility of the formation of shock waves of rarefaction, this work did not use the macroscopic theory of weak shock waves. The BBGKY equations, which at the microscopic level, describe the conditions of thermodynamic and mechanical equilibrium in an arbitrary inhomogeneous system, were put to significant use. Therefore, the results obtained are more general in nature. The phase diagrams showed the areas of implementation of the shock waves of the rarefaction. Hits on the system in such areas can lead to the formation of shock waves. As a result, self-supported shock waves can reach intensities sufficient to dissociate coal molecules with the separation of methyl groups and hydrogen atoms. The subsequent recombination of methyl groups and hydrogen atoms generates significant amounts of methane in outbursts. A prerequisite for the realization of gas-dust outbursts is the presence in the coal array of methane traps with dimensions sufficient to convert the rarefaction wave into a shock and detonation rarefaction wave.

The developed approach was applied to studying the conditions of the autoignition of hydrogen during its sudden release into the atmosphere. The positions of the boundary lines of the self-acceleration of the emission of molecules and the boundary line of self-ignition were calculated on the phase diagram of hydrogen. The phenomenon of a delay in the autoignition of hydrogen during its sharp release into the atmosphere was associated with the time of transition of the hydrogen state from the boundary line of self-acceleration to the state on the boundary line of self-ignition.

High-energy devices using methane and hydrogen at high pressures are widely represented in technology. The phenomena of the self-acceleration of the emission of such gases during the depressurization of such devices can lead to the generation of strong rarefaction shock waves and self-ignition, propagating inward to containers with gas. For example, the use of methane at pressures above 500 bar becomes dangerous in the event of a sharp depressurization (Figure 8) due to the self-reinforcement effect of the rarefaction shock wave. This phenomenon has been observed tens of thousands of times during gas and dust outbursts in coal mines and led to huge losses of life and property.

## 8. Conclusions

The presented article is based on the previously developed microscopic theory of high-energy emission phenomena in condensed systems [1,2]. The key role in the phenomena of ejection of atoms and molecules from the surface of condensed systems is played by the single-particle potential of average forces, which is formed as a result of interparticle interactions and correlations, which significantly depends on the thermodynamic parameters of the system. For some values of the thermodynamic parameters, the one-particle potential acting on individual particles can take on a pushing character near the surface, which corresponds to negative values of the work function of particles.

Based on calculations of the single-particle potential of average forces and the work function of molecules from a sharply unloaded flat surface of liquid methane, a microscopic approach has been developed to explain the energy of molecular-kinetic processes leading to gas and dust emissions in coal mines. The initial thermodynamic conditions in a methane trap during its abrupt unloading, which can lead to the phenomenon of self-acceleration of the emission of molecules and the initiation of a rarefaction shock wave, were investigated.

The necessary conditions for the fluidization of coal and the dissociation of hydrocarbon molecules under the action of a plane rarefaction shock wave as it exits the methane trap into coal were analyzed. The release of methane accompanying emissions significantly exceeding the amount of methane adsorbed in pores and dissolved in coal, is associated with the partial dissociation of hydrocarbon molecules under the action of a shock wave and subsequent energetically favorable molecularization with the formation of methane. The positive energy balance of the process of the shock-wave dissociation of molecules and the subsequent molecularization with the formation of a significant amount of methane leads to the formation of the self-sustaining reactive forces of a detonation wave.

The generation of coal nanoparticles in large quantities observed under the conditions of gas and dust outbursts in coal mines is explained by the phenomenon of the instability of the plane front of rarefaction shock waves entering the coal mass. The nonlinear evolution of liquid methane density fluctuations at the front of the rarefaction shock wave leads to the generation of coal nanoparticles. The minimum sizes of coal nanoparticles are determined by the kinematic viscosity, density of liquid methane, and the velocity of emitted methane molecules, and are represented as $\lambda_{\min}\approx \;2\;Å.$

The molecular–kinetic mechanisms of the emission instability of the liquid surface and the criterion for the self-acceleration of the emission of molecules studied in this work are applied to the study of the conditions for the autoignition of hydrogen during its outflow into the atmosphere. The phenomenon of hydrogen autoignition significantly limits the practical use of hydrogen in technology. The phenomenon of the self-acceleration of the emission of molecules from the surface of a liquid leads to an increase in the velocity of the emitted hydrogen molecules to a level sufficient for the shock dissociation of oxygen molecules. The position of the boundary line of the shock dissociation of oxygen molecules by hydrogen molecules is calculated on the phase diagram of hydrogen. The recombination of the atomic-molecular mixture corresponds to the spontaneous ignition of hydrogen escaping into the atmosphere. The delay in the autoignition of hydrogen in the initial stage of its release into the atmosphere is associated with the time of transition of hydrogen at the front of the rarefaction shock wave from the boundary line of the self-acceleration of the molecular emission to the boundary line of the autoignition in the phase diagram of hydrogen.

The results of this work are of a general nature and can be applied to a wide range of molecular kinetic phenomena in energetic materials (high-energy emission, implosion, shock, and detonation waves). The dangerous phenomena of the transition of shock waves into detonation waves in various materials and fuels can be theoretically investigated using the results of this work.

## Data Availability

Data available in a publicly accessible repository.
